# Identification and Differential Expression of microRNAs in Ovaries of Laying and Broody Geese (*Anser cygnoides*) by Solexa Sequencing

**DOI:** 10.1371/journal.pone.0087920

**Published:** 2014-02-04

**Authors:** Qi Xu, Yang Zhang, Yang Chen, Yi-Yu Tong, Guang-Hui Rong, Zheng-Yang Huang, Rong-Xue Zhao, Wen-Ming Zhao, Xin-sheng Wu, Guo- Bin Chang, Guo-Hong Chen

**Affiliations:** 1 Key Laboratory of Animal Genetics & Breeding and Molecular Design of Jiangsu province, Yangzhou University, Yangzhou, People's Republic of China; 2 Jiangsu High Quality Poultry Engineering Technology Center, Changzhou, People's Republic of China; Harbin Medical University, China

## Abstract

**Background:**

Recent functional studies have demonstrated that the microRNAs (miRNAs) play critical roles in ovarian gonadal development, steroidogenesis, apoptosis, and ovulation in mammals. However, little is known about the involvement of miRNAs in the ovarian function of fowl. The goose (*Anas cygnoides*) is a commercially important food that is cultivated widely in China but the goose industry has been hampered by high broodiness and poor egg laying performance, which are influenced by ovarian function.

**Methodology/Principal Findings:**

In this study, the miRNA transcriptomes of ovaries from laying and broody geese were profiled using Solexa deep sequencing and bioinformatics was used to determine differential expression of the miRNAs. As a result, 11,350,396 and 9,890,887 clean reads were obtained in laying and broodiness goose, respectively, and 1,328 conserved known miRNAs and 22 novel potential miRNA candidates were identified. A total of 353 conserved microRNAs were significantly differentially expressed between laying and broody ovaries. Compared with miRNA expression in the laying ovary, 127 miRNAs were up-regulated and 126 miRNAs were down-regulated in the ovary of broody birds. A subset of the differentially expressed miRNAs (G-miR-320, G-miR-202, G-miR-146, and G-miR-143*) were validated using real-time quantitative PCR. In addition, 130,458 annotated mRNA transcripts were identified as putative target genes. Gene ontology annotation and KEGG (Kyoto Encyclopedia of Genes and Genomes) pathway analysis suggested that the differentially expressed miRNAs are involved in ovarian function, including hormone secretion, reproduction processes and so on.

**Conclusions:**

The present study provides the first global miRNA transcriptome data in *A. cygnoides* and identifies novel and known miRNAs that are differentially expressed between the ovaries of laying and broody geese. These findings contribute to our understanding of the functional involvement of miRNAs in the broody period of goose.

## Introduction

MicroRNAs (miRNAs) are a class of endogenous, small [approximately 18–25 nucleotides (nt)], single-stranded, non-coding RNA molecules that regulate gene expression by promoting translational repression and/or degradation of target mRNAs through binding to their 3’untranslated regions (3’UTRs). Since the first miRNA, lin-4, was identified in *Caenorhabditis elegans* approximately two decades ago [1], tens of thousands of miRNAs have been identified in various multicellular organisms, including humans, flies, nematodes, and plants, and deposited in the miRBase database (http://www.mirbase.org/, Release 18.0, September 2013) [2], [3], [4]. However, miRNAs in the goose have not been reported to date. There is increasing evidence that miRNAs play significant roles in various biological processes, including cell proliferation, differentiation, programmed apoptosis and cell death, morphogenesis of specific organs, and the pathogenesis of human diseases [5]–[10]. The expression of most miRNAs exhibits a spatio-temporal pattern, suggesting that they play specific functions in a variety of processes [11]–[12]. Recent progress in understanding the biology and physiology of small RNAs (sRNA) has provided new and exciting perspectives on the regulation of reproductive function by miRNAs [13]–[14]. A previous study showed that impaired ovarian corpus luteum angiogenesis in *Dicer^d/d^* mice was associated with a lack of miR17-5p and let-7b, which participate in angiogenesis by regulating expression of the antiangiogenic factor tissue inhibitor of metalloproteinase (TIMP) [15]. Recent research also indicates possible regulatory effects of miR-196a on the expression of homebox genes in the newborn ovary that are associated with premature ovarian failure [16]. Bta-miR-143, which has been reported to the most highly expressed miRNA in bovine testis and ovary, participates in pathways associated with reproduction [17]. It is therefore conceivable that miRNAs play an important role in ovarian function.

The goose (*Anas cygnoides*) is a commercially important food that is cultivated widely in China. However, the goose industry has been hindered by strong broodiness and poor egg-laying performance, which is strongly associated with ovary cyclical shinking in broody period. In this study, two sRNA libraries were generated from ovary tissues of laying and broody geese. We integrated the Solexa high-throughput sequencing technique and bioinformatics for sequencing and data processing to compare ovarian miRNA expression profiles between laying and broody goose and identify novel and differentially expressed miRNAs. Our miRNA data and expression profiling will promote better understanding of the functional involvement of miRNAs in the goose ovary.

## Materials and Methods

### Ethics Statement

All animal experiments were reviewed and approved by the Institutional Animal Care and Use Committee of Yangzhou University. Experiments were performed in accordance with the Regulations for the Administration of Affairs Concerning Experimental Animals (Yangzhou University, China, 2012) and Standards for the Administration of Experimental Practices (Jiangsu, China, 2008). All operations were performed according to recommendations proposed by the European Commission (1997), and all efforts were made to minimize suffering.

### Goose Rearing and Sample Preparation

Female Zhedong white geese were selected from 100 geese in the breeding farm of Jiangsu Lihua Animal Husbandry Co. Ltd and were raised according to the farm’s standard practice. During the experiment, geese were fed ad libitum with rice grain supplemented with green grass or water plants whenever possible. The feed was given during the daytime when the geese were released to an open area outside the house. The geese were exposed to natural lighting and temperature throughout this study. Ovarian samples were obtained from three laying geese and three broody geese at 380 days of age. The six geese were anesthetized with sodium pentobarbital and ovarian samples, which comprised the whole ovary including the small and large yellow follicles, were rapidly removed, wrapped in a freezing tube, frozen in liquid nitrogen, and stored at −70°C until needed.

### Construction of Small RNA Libraries and Solexa Sequencing

Total RNA was extracted from ovaries of laying and broody geese using Trizol reagent (Invitrogen, USA) in accordance with the manufacturer's protocol. RNA integrity was confirmed using the 2100 Bioanalyzer (Agilent Technologies). Two sRNA libraries were constructed using homogenized and pooled total RNAs of three individuals for each group (laying and broody). For each group, 10 microg of total RNA was used for library construction with a Small RNA Sample Prep Kit (Illumina, USA) following the manufacturer’s instructions with minor modifications. Briefly, after 15% Tris-Borate-EDTA (TBE) denaturing polyacrylamide gel electrophoresis (PAGE) the 18- to 30-nt fraction of total RNA was excised, purified, and ligated to 3’ and 5’ RNA adaptors using T4 RNA ligase. The adaptor-ligated sRNAs were subjected to RT-PCR with 15 cycles of PCR amplification. The PCR products (approximately 90-bp, corresponding to sRNA + adaptors) were purified on 4% agarose gels to create the libraries. The purified libraries were used directly for cluster generation and sequencing analysis using an Illumina/Solexa G1 sequencer (Shanghai Oebiotech Co. Ltd, China).

### Sequencing Data Analysis and Identification of miRNAs

First, the low-quality reads were filtered to remove reads without the 3’ adaptor, 5’ adaptor-contaminant reads, reads without the insert fragment, reads containing poly(A) stretches, and reads of less than 18 nt. Next, the remaining sequences (clean reads) were mapped to the chicken genome using SOAP (http://soap. genomics.org.cn) with a tolerance of one mismatch to analyze their distribution. The sequences were aligned against known miRNA precursors and mature miRNAs deposited in the miRBase 18.0 to identify conserved miRNAs. The clean reads were compared against the sRNAs (rRNAs, tRNAs, snRNAs, snoRNA, miRNA) deposited in the GenBank and Rfam (http://www.sanger.ac.uk/resources/databases/rfam.html) databases to annotate the sRNA sequences. Because some sRNA tags might map to more than one category we used priority rules to ensure that every unique sRNA was mapped to only one annotation as follows: rRNA etc. (GenBank >Rfam) >known miRNA >repeat >exon >intron).

After identifying the conserved miRNAs, the remaining sequences of the two libraries were aligned with the integrated goose transcriptome to predict novel miRNAs. Potentially novel miRNAs were analyzed in two steps, first using Mireap software and then using Mfold software. The Mireap program was used to analyze structural features of the miRNA precursors to identify all novel miRNA candidates. The resulting structures were retained as novel miRNA candidates only if they met the criteria described by Allen et al [18] and Friedlander et al [19]. The novel goose pre-miRNA sequences were checked using Mfold to predict stem-loop structure (http://mfold.rna.albany.edu). The stem-loop hairpins were considered to be typical only when they fulfilled the following criteria: (1) the number of base pairs in a stem was ≥18 nt; (2) the number of errors in one bulge was ≤18; (3) the secondary structures of the hairpins were stable with a free energy of hybridization less than –20 kcal/mol; (4) the percentage of the miRNA in the stem was ≥80%; (5) the length of the hairpin (up and down stem plus terminal loop) was ≥53 nt; (6) the length of the hairpin loop was ≤22 nt; and (7) the percentage of A and U in the mature miRNA was 30%–70%. Any sequence that satisfied these strict criteria was considered a candidate miRNA precursor.

### Expression of Known miRNAs

We compared the expression of the known miRNAs between the two samples to identify differentially expressed miRNAs. miRNA expression in the two samples was analyzed by Log2-ratio figure and Scatter Plot. The procedure was as follows: (1) The expression of miRNA in the two samples (laying and broody) was normalized to obtain expression of the transcript per million (normalized expression (NE)  =  Actual miRNA count/Total count of clean reads*1,000,000). When the normalized expression of a certain miRNA was zero, we revised its expression value to 0.01. If the normalized expression of a certain miRNA was lower than 1, further differential expression analysis was conducted without this miRNA. (2) We calculated fold-change and P-value from the normalized expression and then generated the log2ratio plot and scatter plot. Fold-change  =  log2 (broodiness ovary-NE/laying ovary-NE).

P-value formula:



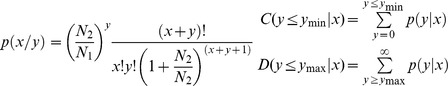



where *N*1 and *x* represent the total number of clean reads and normalized expression level of a given miRNA in the sRNA library generated from the laying ovaries, respectively, and *N*2 and y represent the total number of clean reads and normalized expression level of a given miRNA in the sRNA library generated from broody ovaries, respectively.

### Validation and Expression Analysis of Goose miRNAs

Differentially expressed miRNAs were validated using RT-qPCR. Briefly, miRNA was isolated from the ovaries of laying and broody geese using miRcute miRNA Isolation Kit (TIANGEN, China, DP501), and 3 µL of sRNA was subjected to reverse transcription using the miRcute miRNA First-Strand cDNA Synthesis kit (TIANGEN). The Poly(A) management and RT-PCR reaction conditions were based on the manufacturer’s recommendations. SYBR Green RT-PCR assays were conducted to determine miRNA expression according to the manufacturer’s protocol (primer sequences are shown in Table 1). The PCR temperature profile and reaction conditions were based on the recommendations of the miRcute miRNA qPCR detection kit, and the reactions were performed on an ABI two-step RT-qPCR system (Applied Biosystems 7500, U.S). Amplification was performed using the following cycling parameters: 40 cycles of 94°C for 20 s and 60°C for 34 s. The housekeeping gene U6 served as an internal reference gene. Each sample was analyzed three times. The relative expression of miRNA was calculated using the 2^−ΔΔCt^ method [20]. Independent-sample t-test was used to examine the significance of the differential expression level of each mature miRNA between laying and broody ovary, and the difference was considered significant for P≤0.05.

**Table 1:** Oligonucleotide primers used in the experiments.

Mature miRNA ID	Mature miRNA sequence	Forward primer (5′→3′)
G-miR-320	AAAAGCTGGGTTGAGAGGGCGAA	AAA AGCTGGGTTGAGAGGGCGAA
G-miR-202	TTCCTATGCATATACTTCTT	GCAGCCCCTTCCTATGGATATACTTCTT
G-miR-146	TGAGAACTGAATTCCATATGCGTT	GCCCTGAGAACTGATTTCCAAATGCGTT
G-miR-125b*	ACAAGTCAGGCTCTTGGGAAA	GGGACAAGTCAGGCTCTTGGGAAA
G-miR-143*	AGGTGCAGTGCTGCATCTCT	GGGAGGTGAAGTGCTGCATCTCT
U6		CGCAAGGATGACACGCAAAT

### Predicted Target Genes of Differentially Expressed miRNAs

The putative target sites of miRNA candidates were identified by aligning the miRNA sequences with the integrated goose transcriptome. All predicted target genes conformed to the guidelines suggested by Allen et al [21] and Schwab et al [22], which are as follows: (1) No more than four mismatches between miRNA and target gene (G-U bases count as 0.5 mismatches); (2) No more than two adjacent mismatches in the miRNA/target duplex; (3) No adjacent mismatches in positions 2, 12 of the miRNA/target duplex (5’of the miRNA); (4) No mismatches in positions 10,11 of the miRNA/target duplex; (5) No more than 2.5 mismatches in positions 1,12 of the miRNA/target duplex (5’ of the miRNA); and (6) The minimum free energy (MFE) of the miRNA/target duplex should be ≥75% of the MFE of the miRNA bound to its perfect complement. However, in this study we applied the stricter criterion of no more than two mismatches between the miRNA sequences and the potential miRNA targets.

### Analysis by GO and the KEGG (Kyoto Encyclopedia of Genes and Genomes) Pathway

To better understand miRNA target function and classification, as well as the metabolic regulatory networks associated with goose miRNAs and their targets, we used InterProScan [23] and Blast2go [24] to perform GO annotation and enrichment analysis for three ontologies, molecular function, cellular component, and biological process. The GO terms were significantly enriched in the predicted candidate target genes of the miRNAs and the genes corresponding to certain biological functions. This method maps all target gene candidates to GO terms in the database (http://geneontology.org/) [25], calculates the gene numbers for each term, and applies a hypergeometric test to find significantly enriched GO terms in the target gene candidates compared with the reference gene background. A Bonferroni correction was applied to obtain a corrected P-value. GO terms with corrected P-values ≤0.5 were defined as significantly enriched in the target gene candidates using the following calculation:



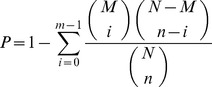



where *N* is the number of all genes with GO annotations; *n* is the number of target gene candidates in N; *M* is the total number of genes that are annotated to a certain GO term; and *m* is the number of target gene candidates in M.

To identify significantly enriched metabolic or signal transduction pathways among the target gene candidates compared with the whole reference gene background we used Cytoscape software V2.8.2 (http://www.cytoscape.org/) [26] and the ClueGO plug-in (http://apps.cytoscape.org/apps/cluego) [27] to decipher the KEGG (http://www.genome.jp/kegg/) [28] pathway and determine biological functions. Genes with FDR ≤0.5 were considered significantly enriched in target gene candidates. The formula used for calculations was the same as that used in the GO analysis.

## Results

### Characteristics and Sequence Analysis of the Small RNAs

**Figure 1:**
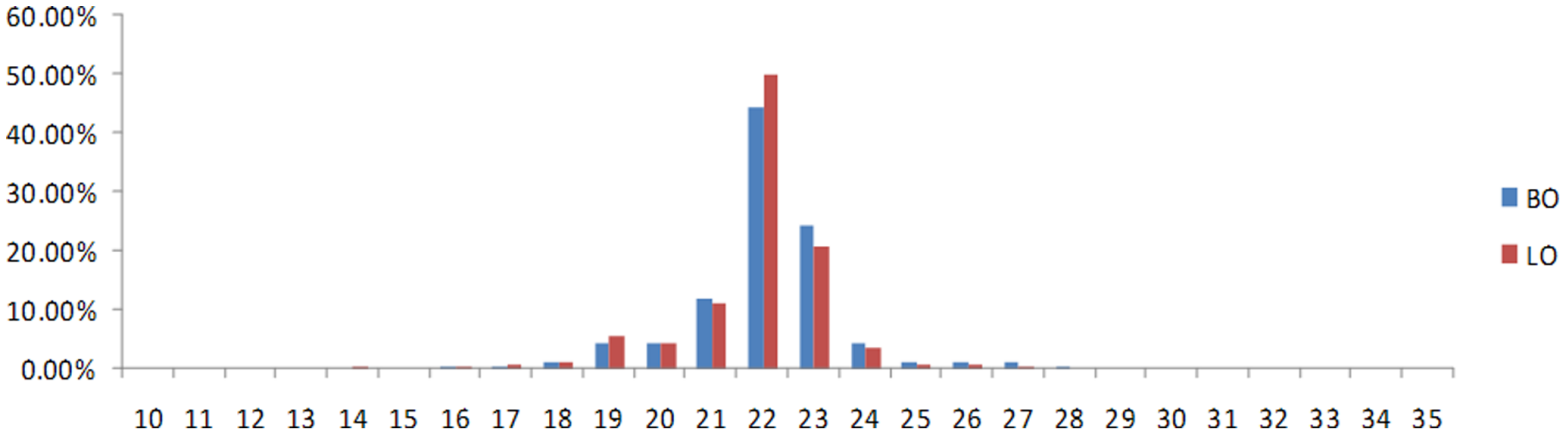
Length distribution for total sRNA reads of the two libraries (BO and LO).

**Figure 2:**
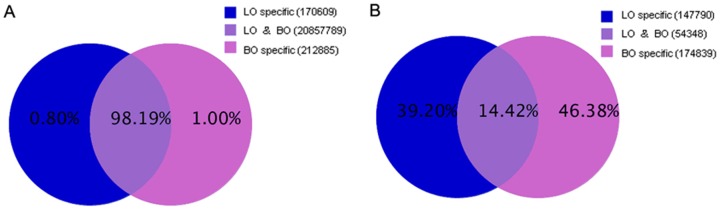
Comparisons of total sRNA reads (A) and unique sRNA reads (B) in the two libraries. The overlapping sector shows common sequences, the other sectors show the respective specific sequences.

After deep sequencing of sRNAs (10 to 30 nt) in the two goose ovary sRNA libraries [laying ovary (LO) and broody ovary (BO)] and removal of the low-quality sequences (reads with low sequencing quality, no 3’ adapter sequence, presence of 5’ adapter sequence, no insert fragment, less than 18 nt, or containing polyA), a total of 11,350,396 and 9,890,887 clean reads were determined for the laying and broody groups, respectively. The length distributions of the total sRNA reads in the two libraries are shown in Figure 1. The majority of sRNAs were 19–24 nt, and the most abundant size class in the sRNA sequence distribution was 22 nt, which accounted for 49.90% and 44.57% of the LO and BO libraries,, respectively, followed by 23 nt (20.85%, 24.29%) and 21 nt (11.00%, 12.12%). Comparison of the total sRNA reads (for sRNAs with more than two reads in the total sRNA reads, only one was included in the analysis), and unique sRNA reads revealed that a large percentage of the total sRNA reads were common to both libraries, whereas the library-specific reads/sequences accounted for only 0.8% to 1.0% of the total sRNA reads (Figure 2). In contrast, only 14.42% of the unique sRNA common sequences were common to both libraries and most of the unique sRNA reads were library-specific (Figure 2).

**Figure 3:**
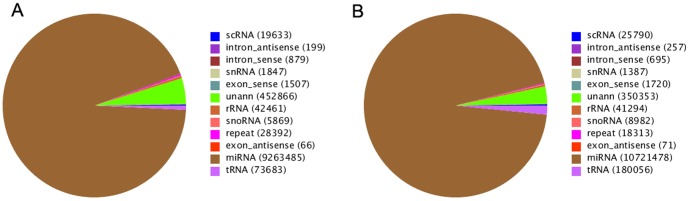
Distribution of sRNAs among different categories in the BO (A) and LO (B) library. The clean reads were annotated and classified as miRNA, rRNA, tRNA, snRNA, snoRNA, etc. based on comparison with GenBank and Rfam databases; partial reads were not annotated.

To assess the efficiency of high-throughput sequencing for sRNA detection, the total population of clean sRNAs were annotated and classified by alignment with GenBank and Rfam databases. The classification annotation revealed that 10,721,478 and 9,263,485 reads in the LO and BO libraries, respectively, were classified as miRNAs, whereas 350,353 and 452,866 reads were unannotated and require further analysis for novel miRNA candidates (Figure 3).

### Identification of Known Conserved miRNAs among Goose miRNAs

To identify known miRNAs in our sequenced set of sRNAs, we compared the sequences recovered from our libraries with the repository of mature miRNAs in miRBase 18.0 using MIREAPv0.2 software. A total of 1,328 conserved miRNAs (1,067 from BO library and 1,088 from LO library) were identified, and 828 of these were present in both libraries (Table S1). However, 501 miRNAs were detected in only one sRNA library. For example, miR-34, miR-129-1*, miR-146b-3p, miR-320c, and miR-125b were only identified in the LO library, whereas miR-129, miR-137-5p, miR-125-5p, miR-129 and miR-147 were present only in the BO library. Some of the known microRNAs belong to the same miRNA family. We obtained a final list of 574 and 493 miRNA families in the LO and BO libraries, respectively.

**Figure 4:**
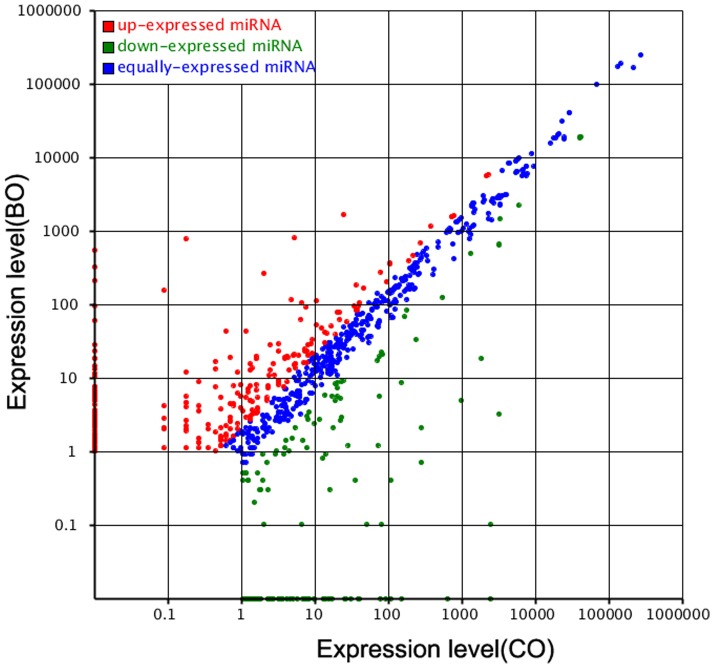
Differential expression of conversed miRNAs between LO and BO library. Each point in the figure represents a miRNA. Red points represent miRNAs with fold-change >2, blue points represent miRNAs with fold-change >1/2 and ≤2, green points represent miRNAs with fold-change ≤1/2.

Comparison of the expression profiles of known miRNAs between the two libraries is shown in Table S2. The expression of known miRNAs was demonstrated using a Log2-ratio and scatter plot (Figure 4). A total of 353 conserved miRNAs were significantly differentially expressed (P<0.01) between the two samples. Compared with miRNA expression in the laying ovary, 127 miRNAs in the broody ovary were significantly up-regulated with P≤0.01, whereas 126 miRNAs were significantly down-regulated with P≤0.01 (Figure 4 and Table S2).

### Identification of Novel microRNA Candidates in Goose

**Figure 5:**
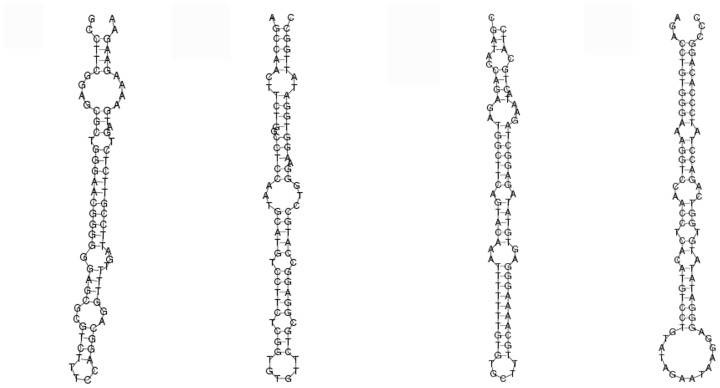
Partial secondary structure of novel microRNAs. Folding secondary structure of novel microRNAs and flanking sequences was predicted by RNAfold. The entire sequence represents pre-miRNAs.

After identifying the conserved miRNAs described above, the remaining sequences of the two libraries were aligned with the goose integrated transcriptome to predict potential novel miRNA candidates. To determine whether these sRNA sequences were genuine goose miRNAs we explored their hairpin structures, Dicer cleavage sites, and minimal free energies using MIREAPv0.2 software (https://sourceforge.net/projects/mireap/) [21]. Mfold [20] and MiPred [24] software were also used to predict the typical secondary structures of the miRNA precursors and remove pseudo-pre-miRNAs. In total, 22 potential novel miRNA candidates with lengths ranging from 20 to 24 nt and reads ranging from 5 to 37 were obtained from LO and BO libraries. These pre-miRNAs possessed a typical stem-loop structure and free energy ranging from –50.8 Kcal/mol to –20.7 Kcal/mol (Table S3). The folding structures of miRNA precursors are shown in Figure 5.

### Validation of Goose miRNAs by qRT-PCR

**Figure 6:**
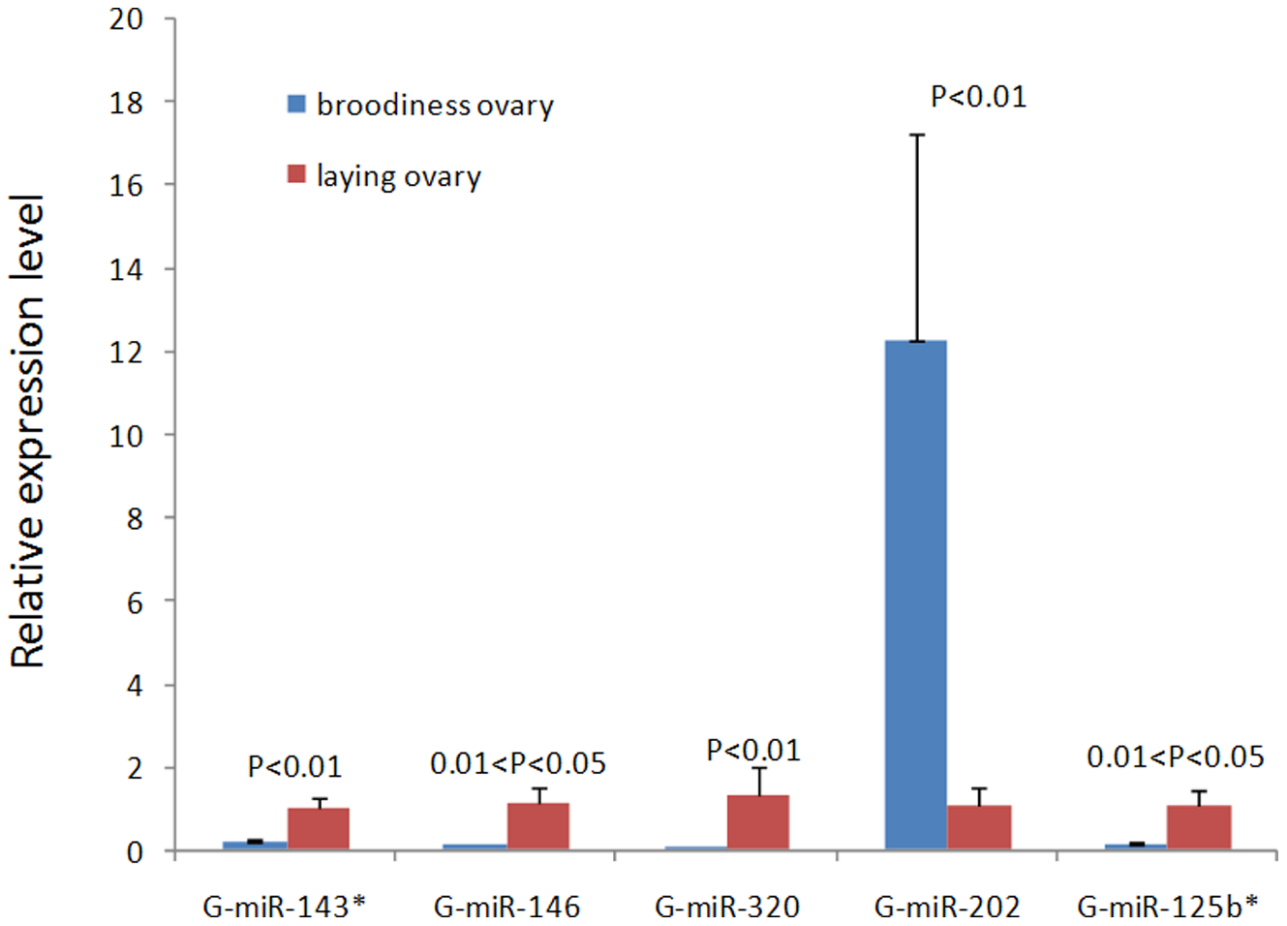
Validation of miRNAs with significantly differential expression using qPCR. Expression levels of five miRNAs were measured by qPCR. The number of biological replicates was three. The data depicted by the Y-axis were calculated using the expression values of 2-ΔΔCt and expressed as means±standard deviation. The significance of differences in expression between samples was calculated by t-test. The corresponding significance value (P) is shown above the respective columns. A P-value between 0.01 and 0.05 was considered significant and P<0.01 was considered highly significant.

To validate the reliability of the sequencing data, we conducted RT-qPCR to compare the expression levels of the differentially expressed miRNAs. We randomly selected five differentially expressed miRNAs (G-miR-320, G-miR-202, G-miR-146, G-miR-125b*, and G-miR-143*) and examined their expression patterns in laying and broody geese. The expression levels of these miRNAs were concordant with their relative reads for Solexa sequencing except for G-miR-125b* (Figure 6). The expression level of G-miR-202 in broody goose was significantly higher than that in laying goose, whereas G-miR-320, G-miR-146, and G-miR-143* were down-regulated in broody goose compared with laying goose.

### miRNA Target Gene Prediction, GO Enrichment, and KEGG Pathway Analysis

To further understand the role of these miRNAs in physiological functions and biologic processes during ovarian atrophy in the goose, miRNA target gene prediction was performed based on miRNA/mRNA interactions to provide some molecular insight into their function. A total of 130,458 annotated mRNA transcripts were predicted as putative target genes for 353 differentially expressed miRNAs (Table S4).

**Figure 7:**
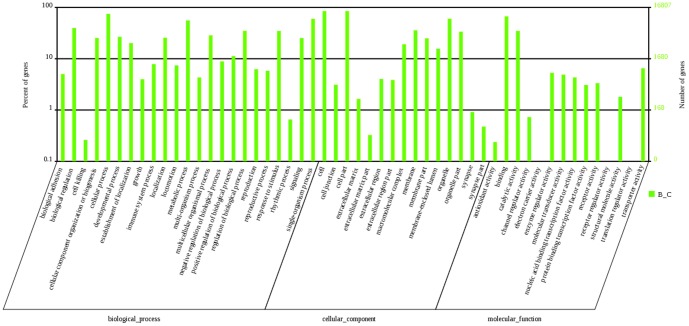
Gene ontology classification annotated by gene2go for target genes of differentially expressed miRNAs. The figure shows partial GO enrichment for the predicted target genes in ontologies of biological processes, cellular component, and molecular function.

The GO enrichment analysis of differentially expressed miRNAs from cellular components showed that 21,962 genes were termed as cellular component ontology with a P-value ≤1. Moreover, 1,936 genes were clustered into “intrinsic to membrane”. Analysis of the molecular function category showed 27,171 genes assigned to different functions although most of the functions were related to binding activity, which had 2,100 annotated genes. Analysis of biological processes showed that 504 genes were involved in hormone secretion biological process and reproduction biological process. Partial GO annotations for predicted target genes are shown in Figure 7.

The predicted target genes were classified according to KEGG function annotations to identify the pathways that were actively regulated by miRNAs in goose ovary tissue (Table S5). KEGG pathway analysis showed 23,889 target genes that were annotated for differentially expressed miRNAs. Most of the target genes were involved in cellular metabolism and signal transduction. The most enriched pathway annotated was “reproductive pathways”, for example TGF-beta signaling pathway, GnRH signaling pathway, and steroid hormone biosynthesis.

## Discussion

miRNAs are a class of small non-coding RNAs that function in gene regulation and play an important role in cell proliferation, maturation, and activity. The regulatory role of these sRNA molecules in the ovary has recently been explored in human [29], mouse [30]–[32], pig [33], cattle [17], [34]–[36], sheep [37] and goat [38]; however, no systematic work has been conducted on the ovary of fowl, including goose. A few ovary miRNAs have been identified by computational and direct cloning approaches [29], [30], [35], but most goose ovarian miRNAs have not been identified or functionally studied. In this study, we created extensive miRNA profiles of ovaries from laying and broody geese. Two sRNA libraries generated a total of 21.2M clean reads, from which 20.4M reads of mappable sequences were derived. Of the mappable sequences, the majority of the sRNAs were 19–24 nt in size, which is typical of the sRNA of Dicer-processed products and similar to that of chicken and other fowl [39]–[41]. In total, 1,328 known conserved miRNAs and 22 novel miRNAs were detected in goose ovary, which will greatly enrich the goose miRBase. In addition, we analyzed differential expression miRNA profiles between laying and broody ovary. The reads of these miRNAs sequences ranged from 1 to 3,085,441, indicating that Solexa sequencing can identify miRNAs with high and low expression. Therefore, Solexa sequencing is a more accurate and efficient approach for studying sRNAs than the traditional cloning method, which only identified 23 miRNAs. Some miRNAs were detected in only one sRNA library, such as miR-34 and miR-129, and some miRNAs showed significantly different expression between the two libraries, such as miR-146 and miR-202, indicating that these miRNAs may have physiological functions in goose ovary tissue.

Because the identification of miRNA candidates was based on the chicken genome sequences (the goose genome has not been sequenced), there may be a few sequence differences in the goose. A total of five conserved miRNAs were randomly selected for RT-qPCR. Four conserved miRNAs were validated; one could not be detected by qRT-PCR, possibly because of inappropriate primer design, very low expression, or because it is a false-positive result, and requires further experimental verification. Of the four validated miRNAs, miR-320 has been extensively studied in the ovary. It was reported that miR-320 is the most abundant miRNA sequence in the newborn ovary [32]. The expression of miR-320 is increased in the ovary of rats with polycystic ovary syndrome [42], and was also found to be significantly up-regulated in TGF-β1-stimulated mouse ovary preantral granulosa cells [43]. This indicates that miR-320 may participate in ovarian function. In addition, miR-202 and miR146 were proven to be associated with reproductive hormone secretion [44]–[45]. A large number of studies have shown that miR-143 (miR-143-3p) might be involved in mammalian reproductive activities [29], [31], [33], [34], [17]. In this study, we found abundant expression of miR-143, which was represented by 185,110 and 283,032 reads in the BO and LO libraries, respectively. However, miR-143 did not show significant differential expression between LO and BO although miR-143*, a member of the miR-143 family, did. Because no 3’UTR database is available it is difficult to predict targets of goose miRNAs. To provide further insight into the physiological functions of miRNAs in goose ovary function, the presumed target genes for the differentially expressed miRNAs were predicted by aligning miRNA sequences to the goose transcriptome. Analysis by GO and KEGG showed that the putative target genes appear to be involved in hormone secretion and reproduction process. These results indicated that some miRNAs might be involved in ovary cell proliferation, apoptosis, and differentiation. Although a large number of target gene candidates were predicted using bioinformatics tools, validation of the relationship between miRNAs and mRNA transcripts requires further experimental evidence.

## Conclusions

In conclusion, in this study we not only obtained the first data for the global transcriptome of goose miRNA but also identified 1,328 conserved known microRNAs and 22 potential novel miRNA candidates. In addition, we demonstrated that some miRNAs (G-miR-320, G-miR-202, G-miR-146, and G-miR-143*) are differentially expressed between ovaries of laying and broody geese. Our integrated analysis provides information that will further our understanding of the functional involvement of miRNAs in the ovary cyclical shinking in the broody period.

### Availability

Illumina sequencing data have been submitted to the Short Read Archive at NCBI and are accessible through accession no. SRP033589.

## Supporting Information

Table S1 **Conserved miRNAs and their expression level in BO and LO libraries.**
(XLS)

Table S2 **Differential expression profile of all known miRNAs in BO and LO libraries.**
(XLSX)

Table S3 **Characteristics of novel miRNA candidates.**
(XLSX)

Table S4 **Target genes for the miRNAs that were differentially expressed between broody and laying geese.**
(XLS)

Table S5 **KEGG Pathway annotations for target genes of differentially expressed miRNAs.**
(XLS)

## References

[1] Lee RC, Feinbaum RL, Ambros V (1993) The C. elegans heterochronic gene lin-4 encodes small RNAs with antisense complementarity to lin-14. Cell 75: 843–854. 8252621 10.1016/0092-8674(93)90529-y

[2] Griffiths-Jones S, Saini HK, van Dongen S, Enright AJ (2008) miRBase: tools for microRNA genomics. Nucleic Acids Res 36: D154–158. 17991681 10.1093/nar/gkm952 PMC2238936

[3] Griffiths-Jones S (2006) miRBase: the microRNA sequence database. Methods Mol Biol 342: 129–138. 16957372 10.1385/1-59745-123-1:129

[4] Griffiths-Jones S, Grocock RJ, van Dongen S, Bateman A, Enright AJ (2006) miRBase: microRNA sequences, targets and gene nomenclature. Nucleic Acids Res 34: D140–144. 16381832 10.1093/nar/gkj112 PMC1347474

[5] Hwang HW, Mendell JT (2006) MicroRNAs in cell proliferation, cell death, and tumorigenesis. Br J Cancer 94: 776–780. 16495913 10.1038/sj.bjc.6603023 PMC2361377

[6] Wahid F, Shehzad A, Khan T, Kim YY (2010) MicroRNAs: synthesis, mechanism, function, and recent clinical trials. Biochim Biophys Acta 1803: 1231–1243. 20619301 10.1016/j.bbamcr.2010.06.013

[7] Song L, Tuan RS (2006) MicroRNAs and cell differentiation in mammalian development. Birth Defects Res C Embryo 78: 140–149. 10.1002/bdrc.20070 16847891

[8] Jay C, Nemunaitis J, Chen P, Fulgham P, Tong AW (2007) MiRNA profiling for diagnosis and prognosis of human cancer. DNA Cell Biol 26: 293–300. 17504025 10.1089/dna.2006.0554

[9] Liu Y, Li M, Ma J, Zhang J, Zhou C, et al (2013) Identification of differences in microRNAs transcriptomes between porcine oxidative and glycolytic skeletal muscles. BMC Mol Biol. 14: 7. 10.1186/1471-2199-14-7 PMC3599761 23419046

[10] Li T, Wu R, Zhang Y, Zhu D (2011) A systematic analysis of the skeletal muscle miRNA transcriptome of chicken varieties with divergent skeletal muscle growth identifies novel miRNAs and differentially expressed miRNAs. BMC Genomics. 12: 186. 10.1186/1471-2164-12-186 PMC3107184 21486491

[11] Fu Y, Shi Z, Wu M, Zhang J, Jia L, et al (2011) Identification and Differential Expression of MicroRNAs during Metamorphosis of the Japanese Flounder (*Paralichthys olivaceus*). PLoS ONE 6: e22957. 21818405 10.1371/journal.pone.0022957 PMC3144956

[12] Chen Q, Lu L, Hua H, Zhou F, Lu L, et al (2012) Characterization and Comparative Analysis of Small RNAs in Three Small RNA Libraries of the Brown Planthopper (*Nilaparvata lugens*). PLoS ONE 7: e32860. 22412935 10.1371/journal.pone.0032860 PMC3295781

[13] Baley J, Li J (2012) MicroRNAs and ovarian function. J Ovarian Res. 5: 8. 10.1186/1757-2215-5-8 PMC3305379 22321819

[14] Donadeu FX, Schauer SN, Sontakke SD (2012) Involvement of miRNAs in ovarian follicular and luteal development. J Endocrinol. 215: 323–334. 10.1530/JOE-12-0252 23038794

[15] Otsuka M, Zheng M, Hayashi M, Lee JD, Yoshino O, et al (2008) Impaired microRNA processing causes corpus luteum insufficiency and infertility in mice. J Clin Invest. 18(5): 1944–1954. 10.1172/JCI33680 PMC2289794 18398510

[16] Bannister SC, Smith CA, Roeszler KN, Doran TJ, Sinclair AH, et al (2011) Manipulation of Estrogen Synthesis Alters MIR202* Expression in Embryonic Chicken Gonads. Biol Reprod 85: 22–30. 21389341 10.1095/biolreprod.110.088476

[17] Huang J, Ju Z, Li Q, Hou Q, Wang C, et al (2011) Solexa sequencing of novel and differentially expressed microRNAs in testicular and ovarian tissues in Holstein cattle. Int J Biol Sci. 7: 1016–26. 10.7150/ijbs.7.1016 PMC3164151 21912509

[18] Allen E, Xie Z, Gustafson AM, Carrington JC (2005) microRNA-directed phasing during trans-acting siRNA biogenesis in plants. Cell 121: 207. 15851028 10.1016/j.cell.2005.04.004

[19] Friedlander MR, Chen W, Adamidi C, Maaskola J, Einspanier R, et al (2008) Discovering microRNAs from deep sequencing data using miRDeep. Nature biotechnology 26: 407–415. 10.1038/nbt1394 18392026

[20] Livak KJ, Schmittgen TD (2001) Analysis of relative gene expression data using real-Time quantitative PCR and the 22ggCT method. Methods 25: 402–408. 11846609 10.1006/meth.2001.1262

[21] Allen E, Xie Z, Gustafson AM, Carrington JC (2005) microRNA-directed phasing during trans-acting siRNA biogenesis in plants. Cell 121: 207–221. 15851028 10.1016/j.cell.2005.04.004

[22] Schwab R, Palatnik JF, Riester M, Schommer C, Schmid M, et al (2005) Specific effects of microRNAs on the plant transcriptome. Dev Cell 8: 517–527. 15809034 10.1016/j.devcel.2005.01.018

[23] Quevillon E, Silventoinen V, Pillai S (2005) InterProScan: protein domains identifier. Nucleic Acids Res 33: 116–120. 10.1093/nar/gki442 PMC1160203 15980438

[24] Conesa A, Gotz S, Garcia-Gomez JM, Terol J, Talon M, et al (2005) B1ast2GO: a universal tool for annotation, visualization and analysis in functional genomics research. Bioinformatics 21: 3674–3676. 16081474 10.1093/bioinformatics/bti610

[25] Carbon S, Ireland A, Mungall CJ, Shu S, Marshall B, et al (2009) AmiGO: online access to ontology and annotation data. Bioinformatics 25: 288–289. 19033274 10.1093/bioinformatics/btn615 PMC2639003

[26] Smoot ME, Ono K, Ruscheinski J, Wang PL, Ideker T (2011) Cytoscape 2.8: new features for data integration and network visualization. Bioinformatics 27: 431–432. 21149340 10.1093/bioinformatics/btq675 PMC3031041

[27] Bindea G, Mlecnik B, Hackl H, Charoentong P, Tosolini M, et al (2009) C1ueG0: a Cytoscape plug-in to decipher functionally grouped gene ontology and pathway annotation networks. Bioinformatics 25: 1091–1093. 19237447 10.1093/bioinformatics/btp101 PMC2666812

[28] Kanehisa M, Goto S, Sato Y, Furumichi M, Tanabe M (2012) KEGG for integration and interpretation of large-scale molecular datasets. Nucleic Acids Res 40: 109–114. 10.1093/nar/gkr988 PMC3245020 22080510

[29] Landgraf P, Rusu M, Sheridan R, Sewer A, Iovino N, et al (2007) A mammalian microRNA expression atlas based on small RNA library sequencing. Cell 129: 1401–1414. 17604727 10.1016/j.cell.2007.04.040 PMC2681231

[30] Ro S, Song R, Park C, Zheng H, Sanders KM, et al. ( 2007) Cloning and expression profiling of small RNAs expressed in the mouse ovary. RNA 13: 2366–2380. 17951331 10.1261/rna.754207 PMC2080611

[31] Mishima T, Takizawa T, Luo SS, Ishibashi O, Kawahigashi Y, et al (2008) MicroRNA (miRNA)cloning analysis reveals sex differences in miRNA expression profiles between adult mouse testis and ovary. Reproduction 136: 811–822. 18772262 10.1530/REP-08-0349

[32] Ahn HW, Morin RD, Zhao H, Harris RA, Coarfa C, et al (2010) MicroRNA transcriptome in the newborn mouse ovaries determined by massive parallel sequencing. Mol Hum Reprod 16: 463–471. 20215419 10.1093/molehr/gaq017 PMC2882868

[33] Li M, Liu Y, Wang T, Guan J, Luo Z, et al (2011) Repertoire of porcine microRNAs in adult ovary and testis by deep sequencing. Int J Biol Sci 7: 1045–1055. 21927574 10.7150/ijbs.7.1045 PMC3174389

[34] Hossain MM, Ghanem N, Hoelker M, Rings F, Phatsara C, et al (2009) Identification and characterization of miRNAs expressed in the bovine ovary. BMC Genomics 10: 443. 19765282 10.1186/1471-2164-10-443 PMC2762473

[35] Tripurani SK, Xiao C, Salem M, Yao J (2010) Cloning and analysis of fetal ovary microRNAs in cattle.Anim Reprod Sci. 120: 16–22. 10.1016/j.anireprosci.2010.03.001 20347535

[36] Miles JR, McDaneld TG, Wiedmann RT, Cushman RA, Echternkamp SE, et al (2012) MicroRNA expression profile in bovine cumulus–oocyte complexes: possible role of let-7 and miR-106a in the development of bovine oocytes. Anim Reprod Sci 130: 16–26. 22269106 10.1016/j.anireprosci.2011.12.021

[37] McBride D, Carre W, Sontakke S, Hogg CO, Law AS, et al (2012) Identification of miRNAs associated with the follicular–luteal transition in the ruminant ovary. Reproduction 144: 221–233. 22653318 10.1530/REP-12-0025

[38] Zhang XD, Ling YH, Zhang YH, Li YS, Liu Ya, et al (2013) MicroRNAs in Ovaries of Goats (*Capra hircus*) Identified by Solexa Sequencing. Scientia Agricultura Sinica 46: 146–153.

[39] Wang XG, Yu JF, Zhang Y, Gong DQ, Gu ZL (2012) Identification and characterization of microRNAs from chicken adipose tissue and skeletal muscle. Poult Sci 91: 139–49. 22184439 10.3382/ps.2011-01656

[40] Yao J, Wang Y, Wang W, Wang N, Li H (2011) Solexa sequencing analysis of chicken pre-adipocyte microRNAs. Biosci Biotechnol Biochem 75: 54–61. 21228487 10.1271/bbb.100530

[41] Zhang L, Nie Q, Su Y, Xie X, Luo W, et al (2013) MicroRNA profile analysis on duck feather follicle and skin with high-throughput sequencing technology. Gene 25: 77–81. 10.1016/j.gene.2013.01.043 23384715

[42] Kong FJ (2011) The expression of microRNA in the ovary of polycystic ovary syndrome rat model, Academic dissertation, Huazhong University of Science and Technology.

[43] Yao GD (2011) microRNA-224 involvement in ovarian follicular development in mouse, Academic dissertation, University of Science and Technology of China.

[44] Bannister SC, Smith CA, Roeszler KN, Doran TJ, Sinclair AH, et al (2011) Manipulation of estrogen synthesis alters MIR202* expression in embryonic chicken gonads. Biol Reprod 85: 22–30. 21389341 10.1095/biolreprod.110.088476

[45] Sirotkin AV, Ovcharenko D, Grossmann R, Lauková M, Mlyncek M (2009) Identification of microRNAs controlling human ovarian cell steroidogenesis via a genome-scale screen. J Cell Physiol 219: 415–420. 19194990 10.1002/jcp.21689

